# Editorial: Pediatric integrated care: from vision to practice

**DOI:** 10.3389/fpsyt.2024.1445148

**Published:** 2024-06-20

**Authors:** Cori M. Green, Cody A. Hostutler, Kathryn L. Lovero, Jennifer A. Mautone, Rheanna Platt

**Affiliations:** ^1^ Department of Pediatrics, Weill Cornell Medicine, Cornell University, New York, NY, United States; ^2^ Department of Pediatric Psychology and Neuropsychology, Nationwide Children’s Hospital, Columbus, OH, United States; ^3^ Department of Pediatrics, The Ohio State University, Columbus, OH, United States; ^4^ Department of Sociomedical Sciences, Mailman School of Public Health, Columbia University, New York, NY, United States; ^5^ Department of Child & Adolescent Psychiatry & Behavioral Sciences, Children’s Hospital of Philadelphia, Philadelphia, PA, United States; ^6^ Department of Psychiatry, Perelman School of Medicine, University of Pennsylvania, Philadelphia, PA, United States; ^7^ Department of Psychiatry and Behavioral Sciences, Johns Hopkins University, Baltimore, MD, United States

**Keywords:** pediatric, integrated care, mental health services, access to mental health care, collaborative care

Globally, mental health disorders and related impairments are the largest contributors to the burden of disease in children and adolescents; with estimates of over 50% of youth meeting criteria for at least one mental health disorder during childhood (1, 2). The COVID-19 pandemic exacerbated an existing youth mental health crisis, highlighting the inadequacy of existing service systems to meet youth mental health needs and broadening already large disparities in access to care (3–6). Pediatric models of integrating mental health services into primary care are effective at preventing and treating mental health conditions (7, 8) and reducing inequities in mental health access and outcomes (9). However, they are less broadly implemented than adult models. Research is needed to understand the factors that contribute to successful implementation and support sustainability to promote more widespread adoption of integrated care in pediatrics.

Too often, the adult evidence-base is used to inform implementation, payment, and policy for pediatric mental health integration. But children are not simply “small adults” and systems of care should be designed specifically to address pediatric needs. The goals of pediatric primary care are often different than adult primary care, including a larger emphasis on prevention, addressing developmental outcomes, and supporting engagement of caregivers (10, 11). Pediatric primary care clinicians often have the unique role of providing care during the onset of mental health disorders with nearly 75% start by age 24 (12, 13). They are typically responsible for monitoring and addressing the progression over time, while considering the intersecting influences of patient development, culture, and the environment (including home and school) (9, 14). Pediatric providers also have opportunities to build wellness and intervene before symptoms become disorders. Therefore, disorder-based models of care cannot be the only answer for pediatric mental health integration; yet, currently policy often focuses on narrow, disease-focused integration models.

Given the combination of increasing mental health needs among youth, increasing interest in integration, and the nascent state of implementation and clinical outcomes in pediatrics, the goals of this Research Topic were to (a) highlight emerging models, as well as their associated implementation and/or clinical outcomes, of pediatric integrated care that can address the unique needs of youth and families; (b) advance our understanding of the specific challenges and facilitators of developing, evaluating, implementing, and sustaining pediatric integrated care models in a range of health system contexts; (c) describe the role of pediatric integrated care within the larger mental health and primary care service system context; and (d) define and assess the components, resources, and policies required to ensure the successful implementation and sustainment of pediatric integrated care. The articles in this collection describe a range of models of integration, settings, and experiences from programs at different levels of development, from conceptualization and piloting to scaling/dissemination. Despite these differences, the articles highlight some common themes, challenges, and areas for future study.

Several studies describe differences in outcomes by patient characteristics. For example, Vanderwood et al. described benefits of the Collaborative Care Model (CoCM) delivered via telehealth to address anxiety and depressive symptoms. They noted lower rates of improvement among patients with depressive symptoms and public insurance relative to those with private insurance, suggesting a need to address additional factors that may influence response to treatment among economically disadvantaged groups. Additionally, Chakawa et al. and Wellen et al. describe the benefits of higher levels of integration among minoritized youth. Wellen et al. found a positive association between same-day, integrated behavioral health consultation and later uptake of mental health services, an effect that was particularly strong for youth with minoritized racial and ethnic identities. Chakawa et al. found that integrated visits within primary care enhanced later patient engagement in co-located or specialty care visits within racial and ethnic groups; however, when compared to their White counterparts, Black youth were least likely to be scheduled for and attend a separate co-located or specialty behavioral health visit following an integrated visit. Of note, although both studies included minoritized youth, only 6% of the Wellen at al. sample were described as Black.

Many of the studies in this series did include *a priori* adaptations to “*traditional”* integration models to expand their reach. For example, both Vanderwood et al. and Parkhurst et al. made adaptations to the CoCM to allow mental health clinicians to serve patients and collaborate with primary care teams from off-site (i.e., by leveraging telehealth services and through development of a regional network of psychotherapy providers offering collaborative care off-site while retaining the registry and care manager that are core features of CoCM). Tengelitsch et al. evaluated the impact of adding integrated behavioral health consultants to a statewide psychiatric consultation line across the state of Michigan. Like Parkhurst et al., Tengelitsch et al. took a regional approach to care and strategically incorporated select components of models matched to the needs of their community and practices.

Other studies outlined the development and refinement of integrated care and consultation models over time. Lee et al. highlighted the importance of engaging service utilizers early and actively to brainstorm components of programming and ensure its usability. Despite these efforts, the authors described variability in program utilization, which they hypothesized may have been related to billing expectations. Similarly, Schweitzer et al. described efforts made to increase critical model elements (e.g., warm handoffs) over time as well as fiscal challenges sustaining integrated care despite high interest in doing so by participating clinics.

In addition to their clinical implications, these findings have significant policy implications. First, several studies highlighted the importance of highly integrated models of care that include same-day access to services, and this may be particularly important for minoritized youth. However, there are financial challenges to funding these models with fee for service approaches, particularly in states that do not allow for medical and behavioral clinicians to bill on the same day for the same diagnosis. Second, these studies demonstrated the need to understand and tailor integration models to meet the unique needs of different populations, regions, and health systems while maintaining evidence-based tenets of integration (15). Meta-analytic reviews in pediatric populations suggest statistical equivalence in clinical effectiveness between different integration models and levels of integration (7, 8). Thus, rather than prioritizing one model over another, policy must consider the broad range of models that exist, the key effective components of integration, and funding innovative and feasible integration models that meet the need of the implementation context. This allows for greater degrees of flexibility in implementation across adult and pediatric care.

In conclusion, this topic area highlights the unique successes and challenges of developing, implementing, studying, and sustaining integrated mental health services within pediatric practices. There are many reasons to be hopeful that mental health integration will be a major factor in addressing the youth mental health crisis, but continued research is needed to inform programming and policy changes supporting adaptable and sustainable models that meet the diverse needs of all patients and families, regardless of sociodemographic background.

## Author contributions

CG: Writing – review & editing, Writing – original draft, Conceptualization. CH: Writing – review & editing, Writing – original draft, Conceptualization. KL: Writing – review & editing, Writing – original draft, Conceptualization. JM: Writing – review & editing, Writing – original draft, Conceptualization. RP: Writing – review & editing, Writing – original draft, Conceptualization.
